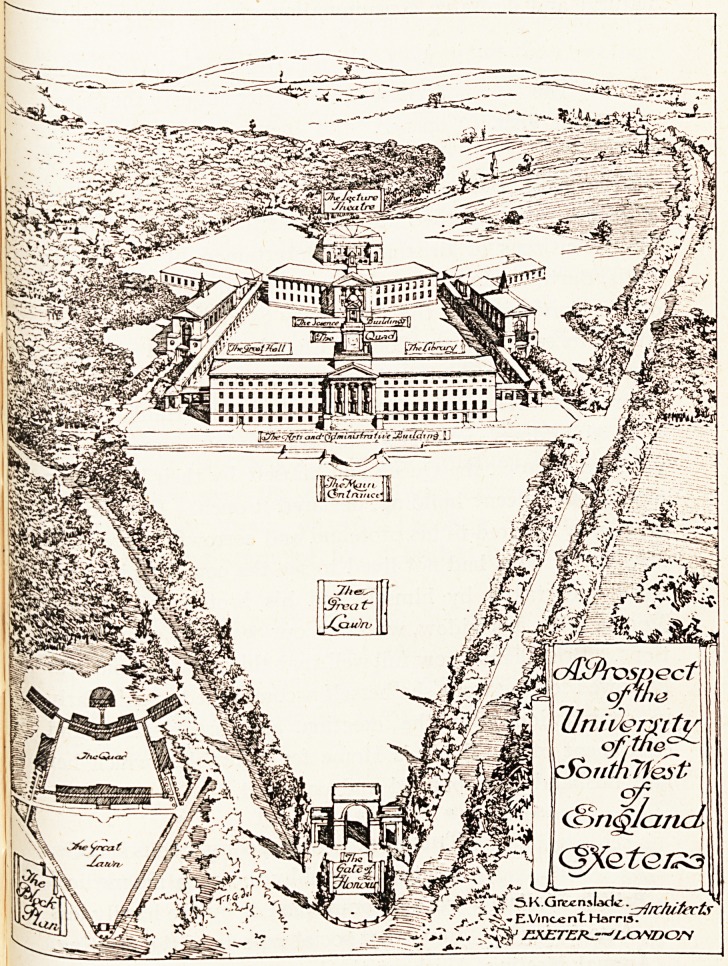# Editorial Notes

**Published:** 1925

**Authors:** 


### Editorial Notes.

**The University College of the South-West.**

THIS College has been in existence for some sixty years, and though founded originally for the service only of the immediate neighbourhood, its activities soon extended to areas much more remote. It continued to grow and develop functions undreamed of by its original founders till, some three years ago, it was recognised by the University Grants Commission as a fully-constituted University College. It now numbers some three hundred and fifty students, and the accommodation of the buildings erected at the beginning of this century is now inadequate to its needs. At the same time the ambitions of the College authorities have likewise expanded, and it is hoped that the new buildings which are to be erected on the Streatham Hall Estate, a recent generous gift to the College, will some day be the home of a federal University of the South-West, with Schools of Medicine, Engineering and Applied Science at Plymouth, Agriculture at Newton Abbot, Mining at Camborne, and Arts and Pure Science at Exeter.

We reproduce the architect's elevation of the proposed new buildings at Exeter: the site is a magnificent one, and the architects have made the most of it. The University College of the South-West is making a modest appeal for £100,000 for new buildings and endowments.

The development of advanced education in arts and science, academic or technical, on the broad lines of the modern University College merits the sympathy and support of the community, and the eventual amalgamation of such

The Lecture Theatre

The Science Buildings

The Quad

The Great Hall

The Library

The Arts and Administrative Building

The Main Entrance

The Great Lawn

The Quad

The Great Lawn

The Block Plan

The Gate of Honour

T.F.G. del

A Prospect of the University of the South West of England Exeter

S.K. Greenslade. E. Vincent Harris. Architects

EXETER and LONDON

centres as constituent colleges of a university is the natural and desirable consummation of these movements.

**The Royal Visit.**

On June 9th His Majesty the King, accompanied by Her Majesty the Queen, opened the new buildings of the University so generously given by Sir George Wills, Bart., and the late Mr. Harry Wills. Their Majesties were accorded an enthusiastic and loyal welcome by the citizens of Bristol, and entertained by Sir George Wills, Bart., the Pro-Chancellor and Chairman of the Council of the University, at the University Students' Union Hall. After proceeding to the Council Chamber of the University, where several presentations were made, the King and Queen entered the Great Hall. The Chancellor, Lord Haldane, read a loyal address, and His Majesty having expressed the pleasure afforded the Queen and himself by their inspection of the magnificent building, declared it open. His Majesty graciously referred to his profound and sorrowful regret that Mr. Harry Wills had not lived to see the completion of the work undertaken by himself and his brother. To Dame Monica Wills, his widow, we offer our warmest congratulations. Bristolians know full well that this great honour was bestowed for the work she herself accomplished, particularly in conjunction with the erection and organisation and equipment of St. Monica's Home for Incurables, which her late husband had founded and endowed.

**The British Medical Association's Annual Meeting at Bath.**

This meeting was a conspicuous success, whether judged by the scientific medical work accomplished or by the hardly less important social aspects of the meeting. Without the hearty co-operation of the Mayor and Corporation, the private hospitality and generous support of

Bath inhabitants and, we may add, of the hotel management, the unstinted efforts of our medical colleagues could never have achieved the distinction attaching to this Bath meeting. Dr. Fred G. Thomson, the President, and Mr. W. G. Mumford, the Honorary General Secretary, on whose shoulders fell the heaviest share in organising the meeting, have every reason to take a legitimate pride in the magnificent results attained.

It was, however, a very great disappointment to everyone that the President's health had not sufficiently recovered from a recent illness to allow of his personal attendance; nevertheless, we had the advantage of hearing his very interesting Presidential Address read by his son, while Mrs. Thomson was a most gracious hostess despite her anxiety.

The most regrettable death of Mr. Forbes Fraser, who would have succeeded to the Presidency of this meeting, and which in fact was due to his initiation, had already cast a shadow over the city, and one cannot doubt that the consequent strain on Dr. Thomson was indirectly the determining cause of his ill-health, in which we all sympathised.

The private and generous hospitality of the Marquis and Marchioness of Bath, the Earl and Countess Temple, the Abbot of Downside, and the President and members of the Bath Division, and of many others were attractive features of this meeting.

**The University of Bristol.**

THE University did honour to the occasion when on the recommendation of Senate honorary degrees were conferred on two of the most distinguished members of the British Medical Association, viz. Sir Humphry Rolleston, Bart., K.C.B., President of the Royal College of Physicians, and Sir Berkeley Moynihan, Bart., K.C.M.G., C.B. Prior to the conferment members of the medical staff entertained at luncheon given in the old Council Chamber of the University the graduates and a large number of medical confrères and their wives from among the members visiting the Bath meeting. After the degree ceremony in the Large Hall the Bristol Branch of the Association received the visitors at tea in the Royal Fort Gardens of the University.

On other days large parties also were taken over the Bristol Savages' home at the Red Lodge, and to Messrs. W. D. and H. O. Wills' and Messrs. J. S. Fry's Factories.

## Figures and Tables

**Figure f1:**